# Identification of three MAPKKKs forming a linear signaling pathway leading to programmed cell death in *Nicotiana benthamiana*

**DOI:** 10.1186/1471-2229-12-103

**Published:** 2012-07-08

**Authors:** Masayoshi Hashimoto, Ken Komatsu, Kensaku Maejima, Yukari Okano, Takuya Shiraishi, Kazuya Ishikawa, Yusuke Takinami, Yasuyuki Yamaji, Shigetou Namba

**Affiliations:** 1 Department of Agricultural and Environmental Biology, Graduate School of Agricultural and Life Sciences, The University of Tokyo, 1-1-1 Yayoi, Bunkyo-ku, Tokyo, 113-8657, Japan

## Abstract

**Background:**

The mitogen-activated protein kinase (MAPK) cascade is an evolutionarily ancient mechanism of signal transduction found in eukaryotic cells. In plants, MAPK cascades are associated with responses to various abiotic and biotic stresses such as plant pathogens. MAPK cascades function through sequential phosphorylation: MAPK kinase kinases (MAPKKKs) phosphorylate MAPK kinases (MAPKKs), and phosphorylated MAPKKs phosphorylate MAPKs. Of these three types of kinase, the MAPKKKs exhibit the most divergence in the plant genome. Their great diversity is assumed to allow MAPKKKs to regulate many specific signaling pathways in plants despite the relatively limited number of MAPKKs and MAPKs. Although some plant MAPKKKs, including the MAPKKKα of *Nicotiana benthamiana* (NbMAPKKKα), are known to play crucial roles in plant defense responses, the functional relationship among MAPKKK genes is poorly understood. Here, we performed a comparative functional analysis of MAPKKKs to investigate the signaling pathway leading to the defense response.

**Results:**

We cloned three novel MAPKKK genes from *N. benthamiana*: *NbMAPKKKβ*, *NbMAPKKKγ*, and *NbMAPKKKε2*. Transient overexpression of full-length NbMAPKKKβ or NbMAPKKKγ or their kinase domains in *N. benthamiana* leaves induced hypersensitive response (HR)-like cell death associated with hydrogen peroxide production. This activity was dependent on the kinase activity of the overexpressed MAPKKK. In addition, virus-induced silencing of *NbMAPKKKβ* or *NbMAPKKKγ* expression significantly suppressed the induction of programmed cell death (PCD) by viral infection. Furthermore, in epistasis analysis of the functional relationships among NbMAPKKKβ, NbMAPKKKγ, and NbMAPKKKα (previously shown to be involved in plant defense responses) conducted by combining transient overexpression analysis and virus-induced gene silencing, silencing of *NbMAPKKKα* suppressed cell death induced by the overexpression of the NbMAPKKKβ kinase domain or of NbMAPKKKγ, but silencing of *NbMAPKKKβ* failed to suppress cell death induced by the overexpression of NbMAPKKKα or NbMAPKKKγ. Silencing of *NbMAPKKKγ* suppressed cell death induced by the NbMAPKKKβ kinase domain but not that induced by NbMAPKKKα.

**Conclusions:**

These results demonstrate that in addition to NbMAPKKKα, NbMAPKKKβ and NbMAPKKKγ also function as positive regulators of PCD. Furthermore, these three MAPKKKs form a linear signaling pathway leading to PCD; this pathway proceeds from NbMAPKKKβ to NbMAPKKKγ to NbMAPKKKα.

## Background

Because plants lack an adaptive immune system, appropriate perceptions and responses of individual cells to various environmental stimuli, such as the biotic stress caused by phytopathogenic microorganisms, are critically important. The plant defense response against biotic stress is triggered by the recognition of conserved pathogen-associated molecular patterns (PAMPs) or of pathogen strain-specific factors known as elicitors or effectors [1]. The response triggered by PAMPs is known as the basal defense response, whereas that triggered by specific elicitors is known as the hypersensitive response (HR). In the latter, an effector is recognized by a corresponding plant resistance (R) protein. The HR is frequently accompanied by programmed cell death (PCD), which plays a particularly important role in the defense against biotrophic pathogens but is also an essential function in normal plant development and differentiation [2]. Although many plant components required for the PCD-associated HR have been identified, the entire signaling pathway leading to PCD has not been elucidated.

The mitogen-activated protein kinase (MAPK) cascade is a highly evolutionarily conserved signal transduction mechanism found in eukaryotic cells. Subsequent to activation of the cascade by various extracellular stimuli, the signal is transduced intracellularly by sequential phosphorylation. In plants, MAPK cascades are associated with developmental and hormonal responses and with stress responses to abiotic and biotic factors [3]. A MAPK cascade consists of three functionally linked protein kinases: a MAPK is phosphorylated and activated by a MAPK kinase (MAPKK), which is in turn activated by an upstream MAPK kinase kinase (MAPKKK). Typical MAPK substrates are cytoplasmic or nuclear proteins, such as transcription factors [3]. MAPKKKs are the most divergent of these three types of kinases in plants; the *Arabidopsis thaliana* genome contains approximately 60 MAPKKKs, 10 MAPKKs, and 20 MAPKs [4].

Based on phylogenetic analysis of the amino acid sequences of their catalytic kinase domains, plant MAPKKKs have been classified into three groups: A, B, and C [4]. Group A contains many MAPKKKs involved in PCD and stress and defense responses; e.g., *A. thaliana* AtMEKK1 is involved in the signaling pathway of basal defense induced by PAMPs [5], and *Medicago sativa* MsOMTK1 [6] is involved in that of oxidative stress-induced cell death. Group A also includes MAPKKKs that have important functions in HR induction. Silencing of the genes encoding *Nicotiana tabacum* NPK1 (NtNPK1) and *Nicotiana benthamiana* MAPKKKα (NbMAPKKKα) suppresses the *N* gene-mediated HR induced by the helicase domain of tobacco mosaic virus (TMV) replicase and *Pto*-mediated HR induced by *Pseudomonas syringae* pv. tomato (Pst) effector avrPto, respectively [7,8]. Recently, *N. benthamiana* NbMAPKKKε and its tomato (*Solanum lycopersicum*) ortholog SlMAPKKKε have been implicated in PCD induction in the HR against Gram-negative bacterial pathogens [9]. In addition, silencing of the genes encoding the MAPKK MEK2 and the MAPK SIPK, both of which act downstream of NbMAPKKKα, also attenuates the *N* gene-mediated HR against TMV [10]. Conversely, silencing of the tomato orthologs of MAPKK MEK1 and MAPK NTF6, both of whose tobacco orthologs act downstream of NtNPK1in tobacco, leads to loss of the *Pto*-mediated HR in tomato [11]. Therefore, the NtNPK1- and NbMAPKKKα-initiated MAPK cascades are essential for both the *N* gene-mediated and the *Pto*-mediated HR, suggesting that at least two distinct MAPK cascades are involved in the regulation of a single HR event [8]. Furthermore, it is now becoming apparent that two distinct MAPK cascades are involved in non-HR environmental responses [12].

Plants generally appear to use the same MAPKK/MAPK sets in different responses to environmental stimuli. The *A. thaliana* MAPKKs AtMKK4 and AtMKK5 and/or their downstream component MPK6 are involved not only in the signaling pathway for basal defense downstream of AtMEKK1 but also in ethylene production and stomata formation [5,13,14]. Given the relatively limited number of MAPKKs and MAPKs in plants, the diversity of these responses (functions) is assumed to be possible due to the great diversity of MAPKKKs [15,16]. Therefore, comparative functional analysis among MAPKKKs is needed to reveal the molecular mechanisms underlying a variety of responses to environmental stresses.

We previously showed that systemic necrosis, the disease symptom caused by plantago asiatica mosaic virus Li1 (PlAMV-Li1), was accompanied by resistance traits similar to HR. Using tobacco rattle virus (TRV)-based virus-induced gene silencing (VIGS) [17], we demonstrated that NbSGT1 and NbRAR1, which are important in the HR, and the MAPK cascade including NbMAPKKKα/NbMEK2, are essential for the induction of PCD-associated systemic necrosis induced by PlAMV-Li1 [18,19]. This result and those described above led us to hypothesize that other MAPKKK genes in addition to NbMAPKKKα are involved in the systemic necrosis induced by PlAMV-Li1.

In the present study, we isolated three novel group A MAPKKK genes from *N. benthamiana*, a model plant of the family Solanaceae, using an expressed sequence-tag (EST) database. The three cloned genes were designated *NbMAPKKKβ*, *NbMAPKKKγ*, and *NbMAPKKKε2*. Further study revealed that NbMAPKKKβ and NbMAPKKKγ are positive regulators of PCD. In addition, the results of epistasis analysis performed using VIGS and agroinfiltration suggest that two of these MAPKKKs (NbMAPKKKβ and NbMAPKKKγ), together with NbMAPKKKα, comprise a linear signaling pathway important in the induction of PCD.

## Results

### Cloning of three novel group A MAPKKK genes from *Nicotiana benthamiana*

To conduct a comparative analysis of the roles of MAPKKK genes in defense responses, we first sought to clone *N. benthamiana* MAPKKK genes belonging to group A. Group A includes four subgroups, A1–A4, and contains many genes involved in plant defense responses. To obtain one *N. benthamiana* MAPKKK gene homolog from each group A subgroup, we selected the following: the *Arabidopsis* MAPKKK genes *AtMEKK1*[20], *AtMAPKKKγ*, and *AtMAPKKKε1*[21,22] as representatives of the A1, A2, and A4 subgroups, respectively. For the A3 subgroup, which includes NtNPK1 [23] and AtANP1, we were able to amplify *N. benthamiana* cDNA fragments using *NtNPK1*-specific primers; the amplified gene turned out to be 98.0% identical in nucleotide sequence to that encoding the NtNPK1 kinase domain, which has been well characterized for its role in defense responses [7]. Therefore, the A3 subgroup was excluded from further study.

Using the nucleotide sequences encoding the highly conserved kinase domains of AtMEKK1 [19], AtMAPKKKγ, and AtMAPKKKε1 as queries for BLAST searches against the *N. benthamiana*, *N. tabacum*, and *Solanum lycopersicum* EST databases, we obtained three EST sequences: *N. benthamiana* TC15397, *N. tabacum* BP133312, and *S. lycopersicum* BI931567, respectively. Based on these sequences, we designed specific primers to isolate full-length *N. benthamiana* MAPKKK cDNA clones, as described in the Materials and Methods. The cloned *N. benthamiana* MAPKKK genes are predicted to encode proteins with highly conserved kinase domains and more divergence in other regions, a general feature of plant MAPKKK genes [4].

**Figure 1:**
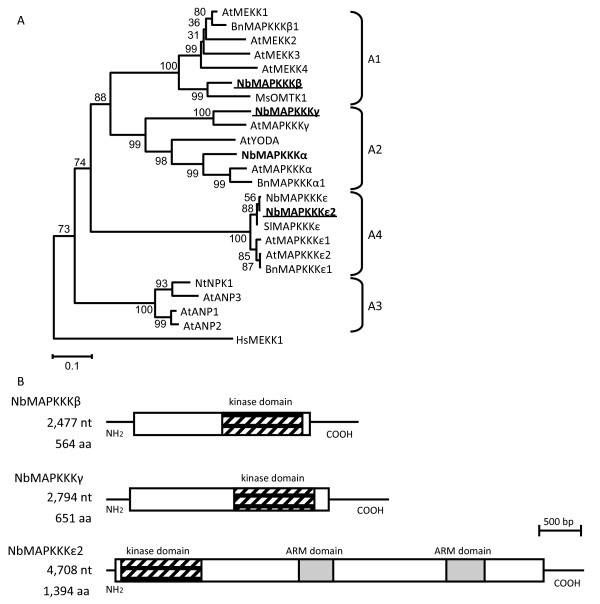
**Phylogenetic analysis of plant MAPKKK genes and structural features of novel *N. benthamiana *MAPKKK genes. A**) Phylogenetic tree for the kinase domain-encoding regions of group A MAPKKK genes. Numbers represent bootstrap scores. Names of genes used in this study are shown in boldface. Names of the three novel MAPKKK genes are underlined. **B**) Diagram of the domain structures of the three novel MAPKKK genes. ORFs are indicated by boxes. The 5′- and 3′-untranslated regions (UTRs) are shown as horizontal lines at the left and right, respectively, of the boxes. Shaded boxes and gray boxes indicate kinase domains and ARM domains, respectively. ORFs and UTRs are drawn to the same scale.

Next, we used the amino acid sequences of the kinase domains of our newly cloned MAPKKKs and those of other previously identified MAPKKKs to construct a phylogenetic tree (Figure 1A). Our newly cloned MAPKKK genes were tentatively designated after the name of the most closely related genes in the phylogenetic tree. Thus, our A2 *MAPKKK* homolog was designated *NbMAPKKKγ* after *A. thaliana AtMAPKKKγ*. At a late phase of this study, an A4 *MAPKKK* homolog, *N. benthamiana NbMAPKKKε*, was reported [9]. Although our A4 homolog shares high sequence similarity with *NbMAPKKKε*, the genes were not identical (96.6% or 95.0% identity at the nucleotide or amino acid level, respectively) and differed in length. Because these results indicated that A4 *MAPKKK* homologs in *N. benthamiana* consisted of at least two genes, our A4 homolog was designated *NbMAPKKKε2* after *NbMAPKKKε*. In fact, from the recently released *N. benthamiana* draft genome sequence (http://solgenomics.net/), only two contigs that show high sequence homology (about ~90%) with *NbMAPKKKε2* were retrieved and each of these sequences corresponded to *NbMAPKKKε2* and *NbMAPKKKε*, respectively. Although our A1 *MAPKKK* homolog was most closely related to *MsOMTK1*, which was designated after the functional features of its gene products, designating this gene “*NbOMTK1*” was apparently incorrect, as we do not know if our A1 homologous gene product and MsOMTK1 have a similar function. The next most closely related genes were *AtMEKK1* and *Brassica napus BnMAPKKKβ1*. Therefore, our A1 homolog was designated *NbMAPKKKβ* after *BnMAPKKKβ1*, a gene of the A1 subgroup, in accordance with other newly cloned genes *NbMAPKKKγ* and *NbMAPKKKε2*. The *NbMAPKKKβ*, *NbMAPKKKγ*, and *NbMAPKKKε2* cDNA sequences determined in this study were deposited in the DNA Data Bank of Japan (DDBJ) under the accession numbers AB649283, AB649284, and AB649285, respectively.

The domain structures of the three novel MAPKKK genes are shown in Figure 1B. The *NbMAPKKKβ*, *NbMAPKKKγ*, and *NbMAPKKKε2* cDNA are 2477, 2794, and 4708 bp in length, respectively, and are respectively predicted to encode proteins of 564, 651, and 1395 amino acids. Kinase domains are found in the C-terminal regions of NbMAPKKKβ and NbMAPKKKγ and the N-terminal region of NbMAPKKKε2. The C-terminal region of NbMAPKKKε2 contains two ARM (armadillo/β-catenin-like repeat) domains. The domain structures of these proteins are similar to those of their respective *A. thaliana* homologs.

### Overexpression of the NbMAPKKKβ and NbMAPKKKγ kinase domains, but not the NbMAPKKKε2 kinase domain, causes cell death

**Figure 2:**
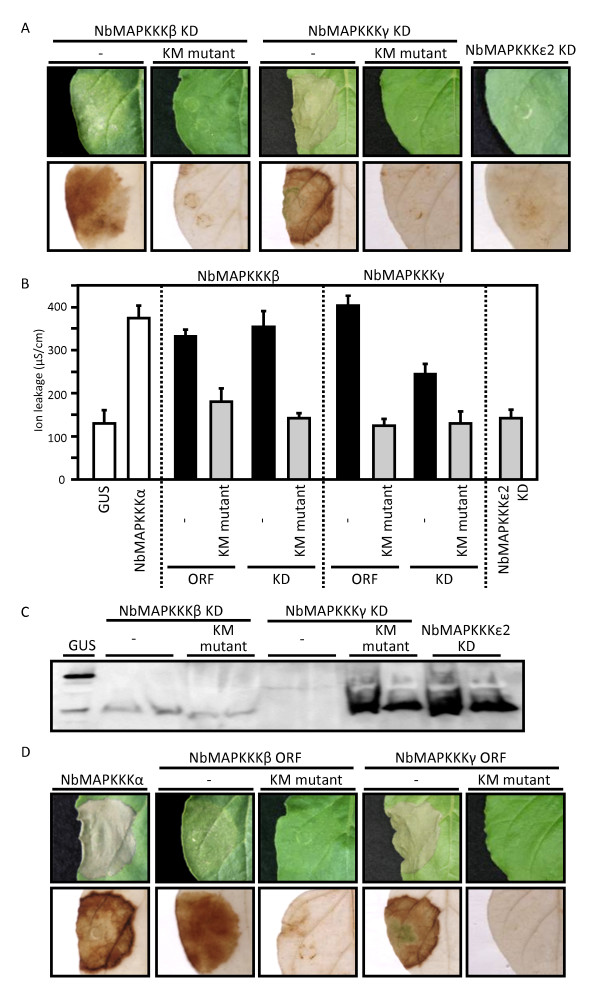
**Overexpression of NbMAPKKKβ and NbMAPKKKγ induces pathogen-independent cell death in *N. benthamiana *leaves. ***N. benthamiana * leaves were infiltrated with *Agrobacterium * strains carrying pEarleyGate203 vector [32] derivatives harboring the coding sequences for mutant or full-length MAPKKKs. All experiments, except that shown in Figure 4, were repeated at least three times with similar results. **A**) Symptoms of infiltrated *N. benthamiana* leaf areas overexpressing the indicated MAPKKK kinase domain (KDs) or ATP-binding site-deficient (K → M) KD mutants. Images of the same leaves after DAB staining are shown in the lower panels. DAB staining to detect hydrogen peroxide production was performed as previously described [18]. Pictures were taken 5 days post-infiltration (dpi). Each protein was transiently co-expressed with silencing suppressor p19. *Agrobacterium* cultures were grown to a turbidity (OD_600_) of 0.5 for use in agroinfiltration. **B**) Ion leakage in the infiltrated leaf areas overexpressing wild-type or K → M mutant NbMAPKKKβ or NbMAPKKKγ or their wild-type or K → M mutant KDs or NbMAPKKKε2 KD. GUS and NbMAPKKKα were used as internal controls. The ion leakage assay was performed as previously described [18]. Data shown represent means ± standard deviation of at least three independent plants. **C**) Western blot analysis of Myc-tagged NbMAPKKKβ or NbMAPKKKγ KDs or their K → M mutants or NbMAPKKKε2 KD. Myc-tagged GUS was used as an internal control. Total proteins were extracted from each gene-infiltrated area at 5 dpi. Two replicates are shown for each Myc-tagged construct. **D**) Symptoms of infiltrated leaf areas overexpressing full-length wild-type or K → M mutant NbMAPKKKβ or NbMAPKKKγ. Images of the same leaves after DAB staining are shown in the lower panels.

Overexpression of the kinase domain of the tomato homolog of NbMAPKKKα, SlMAPKKKα, in *N. benthamiana* leaves by agroinfiltration has been reported to induce pathogen-independent cell death [8]. To investigate whether our three newly identified MAPKKKs also possessed cell death-inducing activity, we performed transient overexpression analysis of each kinase domain using agroinfiltration. The transient overexpression of the kinase domains of NbMAPKKKβ and NbMAPKKKγ induced pathogen-independent cell death in the infiltrated area (Figure 2A). In the infiltrated areas, cell death was associated with significant hydrogen peroxide production, which is detected by a characteristic brown color that emerges upon 3,3'-diaminobenzidine (DAB) staining. These results were confirmed by measuring the extent of cell death using an ion leakage assay (Figure 2B), which showed significantly increased ion leakage in the NbMAPKKKβ- and NbMAPKKKγ-overexpressing areas. No significant difference in the level of ion leakage was observed for NbMAPKKKβ vs. NbMAPKKKγ. In contrast, overexpression of the kinase domain of NbMAPKKKε2 failed to induce cell death or hydrogen peroxide production and failed to increase the level of ion leakage. Western blot analysis using anti-Myc monoclonal antibody (Millipore, Billerica, MA, USA) was performed to assess the expression levels of these kinase domains (Figure 2C). Specific signals of NbMAPKKKβ and NbMAPKKKε2 kinase domains were detected, but no accumulation of NbMAPKKK*γ* kinase domain was found. However, we suppose that the NbMAPKKK*γ* kinase domain was accurately expressed, even at very low levels, because its overexpression rapidly induced cell death. Western blot analysis also showed that the accumulated expression level of NbMAPKKKε2 kinase domain, whose overexpression did not induce cell death, was much greater than the expression levels of the NbMAPKKKβ and NbMAPKKKγ kinase domains. Because these results indicated that NbMAPKKKε2 is not involved in the induction of pathogen-independent cell death, we excluded NbMAPKKKε2 from further analysis.

### Overexpression of full-length, catalytically active NbMAPKKKβ or NbMAPKKKγ causes cell death

To further investigate the involvement of NbMAPKKKβ and NbMAPKKKγ in pathogen-independent cell death, we conducted transient overexpression experiments using their full-length open reading frames (ORFs). As shown in Figure 2D, overexpression of NbMAPKKKβ or NbMAPKKK*γ* induced cell death. The observed level of ion leakage was similar for overexpression of NbMAPKKKβ, NbMAPKKKγ, and NbMAPKKKα, which was used as a positive control (Figure 2B).

Several previous studies have demonstrated that kinase activity is required for the triggering of pathogen-independent cell death by transient overexpression of a component of a MAPK cascade [8,24,25]. To examine whether NbMAPKKKβ- and NbMAPKKKγ-induced cell death also required kinase activity, we constructed full-length and kinase domain MAPKKK mutants deficient in ATP binding. In these mutants, the essential conserved lysine residue (K) in the ATP-binding site of the kinase domain was replaced with a methionine (M) [8]. As shown in Figure 2A and 2D, none of these K → M mutants induced cell death when overexpressed. This result was confirmed by DAB staining (Figure 2A and 2D) and ion leakage assays (Figure 2B). Also, the expression of these kinase domain K → M mutants was confirmed by western blot analysis with anti-Myc monoclonal antibody (Figure 2C). These results indicate that cell death induced by the transient overexpression of *N. benthamiana* NbMAPKKKβ or NbMAPKKKγ is dependent on their kinase activities.

### Silencing of *NbMAPKKKβ* or *NbMAPKKKγ* suppresses PlAMV-Li1-induced PCD

**Figure 3:**
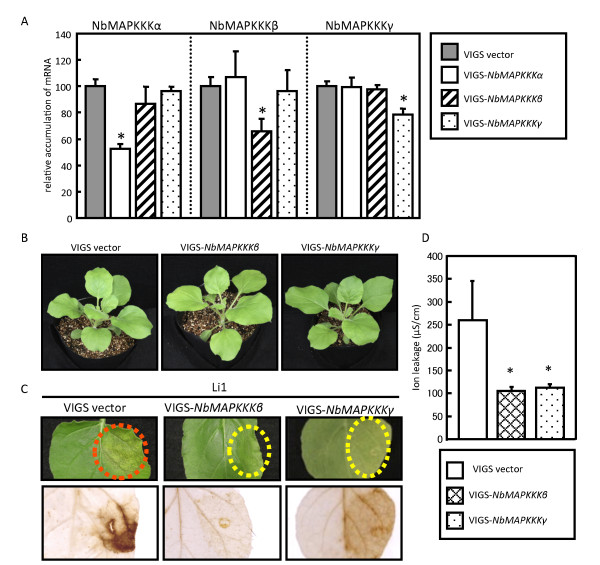
**Silencing of *NbMAPKKKβ *and *NbMAPKKKγ *suppresses PlAMV-Li1-induced PCD. A**) Confirmation of specific MAPKKK gene silencing in gene-silenced plants. The relative quantities of *NbMAPKKKα*, *NbMAPKKKβ*, and *NbMAPKKKγ* mRNA in non-silenced or *NbMAPKKKα*-, *NbMAPKKKβ-*, or *NbMAPKKKγ*-silenced plants were measured using real-time RT-PCR. The data for each leaf tissue sample were normalized to the Nb18S rRNA gene expression level in the same sample. Data shown represent means ± standard deviation of at least three independent plants. Asterisks indicate statistically significant differences from control plants (*P* < 0.05). **B**) Typical phenotypes observed in non-silenced and *NbMAPKKKβ*- and *NbMAPKKKγ*-silenced plants. Pictures were taken 21 dpi. **C**) Symptoms in the PlAMV-Li1-infiltrated areas of non-silenced and *NbMAPKKKβ*- or *NbMAPKKKγ*-silenced plants (upper panels) and DAB staining of the same leaves (lower panels). Red circles indicate cell death; yellow circles indicate no symptoms. Leaves were infiltrated with a PlAMV-Li1-expressing *Agrobacterium* culture grown to a turbidity (OD_600_) of 0.05. **D**) Ion leakage in the PlAMV-Li1-infiltrated areas of non-silenced and *NbMAPKKKβ*- or *NbMAPKKKγ*- silenced plants. Data shown represent means ± standard deviation of at least three independent plants. Asterisks indicate statistically significant differences from control plants (*P* < 0.05).

In light of the above finding that overexpression of full-length NbMAPKKKβ or NbMAPKKKγ or their kinase domains can induce cell death, we expected that silencing of the genes encoding these MAPKKKs would suppress virally induced PCD. Therefore, we used a TRV-based VIGS system [17] to silence *NbMAPKKKβ* and *NbMAPKKKγ*. Successful silencing of each gene was confirmed by analyzing the expression of *NbMAPKKKβ*, *NbMAPKKKγ*, and *NbMAPKKKα* (control) using real-time reverse transcription (RT)-PCR analysis (Figure 3A). Also, Southern blot analyses using a kinase domain-specific probe of each gene revealed that *NbMAPKKKβ* and *NbMAPKKKγ* exist as single-copy and multiple-copy genes, respectively, in the *N. benthamiana* genome (Additional file 1: Figure S1). In the *N. benthamiana* draft genome, two contigs showing high sequence homology (about >90%) with *NbMAPKKKγ* were obtained, suggesting that there are at least two copies of *NbMAPKKKγ* homologs. Thus, we assume that a single gene is specifically targeted in *NbMAPKKKβ*-silenced plants, but one or more highly similar genes might be targeted in *NbMAPKKKγ*-silenced plants. Hereafter, we use the term “*NbMAPKKKγ*” to indicate *NbMAPKKKγ* and/or its homologous gene(s) except for transient overexpression experiments. The gene-silenced plants did not display any obvious phenotypic differences compared with non-silenced (VIGS vector) control plants (Figure 3B), suggesting that neither NbMAPKKKβ nor NbMAPKKKγ is involved in normal plant growth and development. In contrast, silencing of NtNPK1, a MAPKKK involved in cell plate formation in plant cytokinesis and in *N* gene-mediated HR cell death, causes severe stunting of plants [7].

*NbMAPKKKβ*- and *NbMAPKKKγ*-silenced plants and control non-silenced plants were subsequently inoculated with PlAMV-Li1, which induces PCD-associated necrosis in *N. benthamiana*. In the PlAMV-Li1-infiltrated areas of non-silenced plants, we observed characteristic necrotic symptoms and brown color staining, indicating the accumulation of hydrogen peroxide. However, in the Li1-infiltrated *NbMAPKKKβ*-silenced plants, cell death and hydrogen peroxide production were completely compromised. Also, in *NbMAPKKKγ*-silenced plants, cell death and hydrogen peroxide production were suppressed completely and partially, respectively, despite only about 20% decrease in the abundance of *NbMAPKKKγ* transcripts (Figure 3C). Suppression of cell death in the *NbMAPKKKβ*- and *NbMAPKKKγ*-silenced plants was confirmed using ion leakage assays (Figure 3D). The suppression of cell death in *NbMAPKKKγ*-silenced plants despite the slight decrease in its mRNA can be explained by a strict requirement of a high level of NbMAPKKKγ protein in cell death. Although it is possible that residual NbMAPKKKγ protein contributes to the decreased level of hydrogen peroxide production, its level might be too low to induce cell death. These results indicate that NbMAPKKKβ and NbMAPKKKγ are involved in hydrogen peroxide production and PCD induced by PlAMV-Li1.

### NbMAPKKKα, NbMAPKKKβ, and NbMAPKKKβ form a linear signaling pathway that induces cell death

Together with the findings of our previous study showing that NbMAPKKKα is involved in PlAMV-Li1-induced PCD [19], the above findings demonstrate that *NbMAPKKKβ* and *NbMAPKKKγ* are also essential for this virally induced PCD. To determine the relationships among these three genes in the PCD signaling pathway, we designed epistasis experiments combining transient overexpression and silencing of combinations of these genes. In these experiments, plants with a specific VIGS-silenced MAPKKK gene were agroinfiltrated with an *Agrobacterium* culture expressing a different MAPKKK gene, and the level of ion leakage was measured. Prior to the epistasis experiments, a preliminary experiment was performed to determine the minimum turbidity of *Agrobacterium* inoculum for each gene sufficient to induce complete cell death. Complete cell death was observed at the following cell densities at OD_600_: 0.5, NbMAPKKKα; 1.0, NbMAPKKKβ; and 0.05, NbMAPKKKγ. Although each MAPKKK was overexpressed at different concentrations of *Agrobacterium* inocula, no significant difference in the basal ion leakage level was observed among the different turbidities (ranging from 0.05 to 1.0) of *Agrobacterium* inocula that expressed the GUS gene in wild-type plants (Additional file 2: Figure S2). For NbMAPKKKβ, cell death induced by both the full-length ORF and kinase domain was slow and weak, but cell death induced by the kinase domain was more often observed than that induced by the full-length ORF. In the ion leakage assay shown in Figure 2B, this tendency was detected, although the difference in cell death induced by the full-length ORF and kinase domain of NbMAPKKKβ was not statistically significant. Previous reports have shown that the substrate specificity of MAPKKK is not affected when only the kinase domain is transiently activated [5,8,26]. Hence, the kinase domain of NbMAPKKKβ was used for further analysis, instead of the full-length ORF.

**Figure 4:**
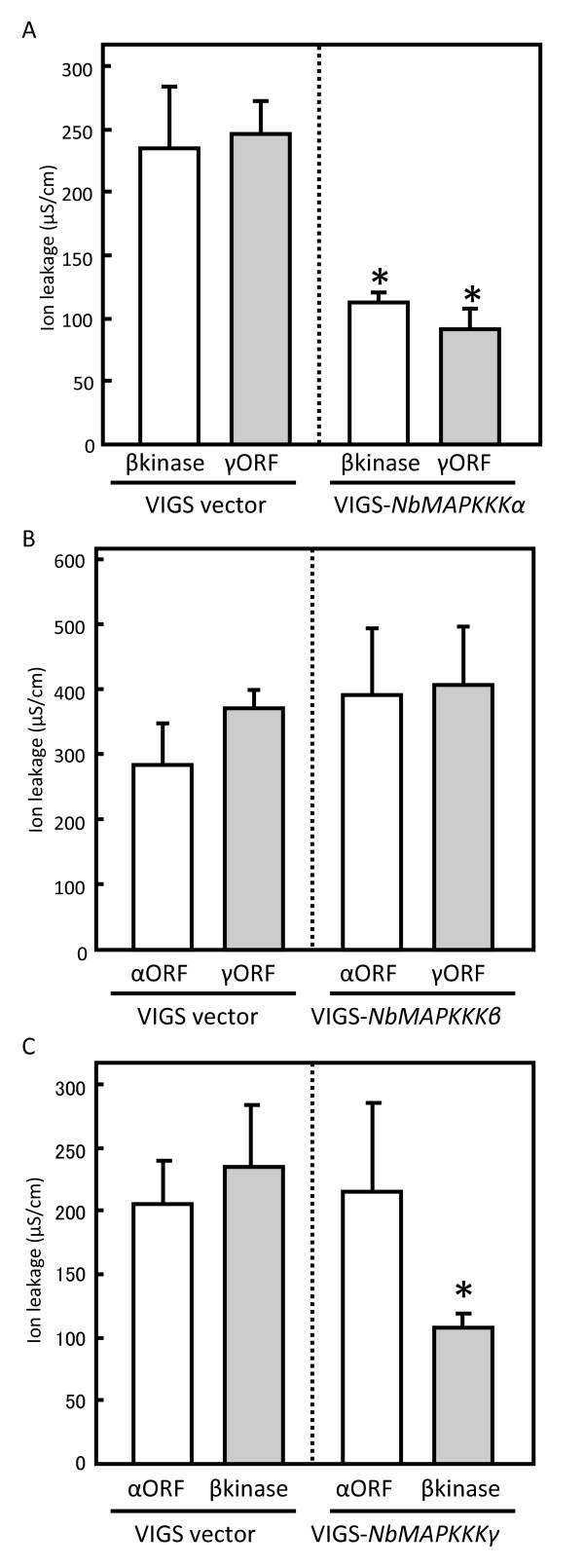
**Epistasis analysis of the functional relationships among NbMAPKKKα, NbMAPKKKβ, and NbMAPKKKγ.** Epistasis analysis was performed by combining functional activation and suppression of specific combinations of MAPKKKs using transient overexpression and VIGS, respectively. Cell death in infiltrated areas was quantified using ion leakage assays at 4 dpi. Data shown represent means ± standard deviation for at least five plants. Asterisks indicate statistically significant differences from control plants (*P* < 0.05). All experiments described in this figure were repeated two times with similar results. **A**) Cell death in the NbMAPKKKβ kinase domain (βkinase)- or NbMAPKKKγ (γORF)-overexpressing areas of non-silenced and *NbMAPKKKα*-silenced plants. *Agrobacterium* cultures expressing βkinase and γORF were grown to a turbidity of 1.0 and 0.05, respectively, for infiltration. **B**) Cell death in the NbMAPKKKα (αORF)- or γORF-overexpressing areas of non-silenced and *NbMAPKKKβ*-silenced plants. *Agrobacterium* cultures expressing αORF and γORF were grown to a turbidity of 0.5 or 0.05, respectively, for infiltration. **C**) Cell death in the αORF- or βkinase-overexpressing areas of non-silenced and *NbMAPKKKγ*-silenced plants. *Agrobacterium* cultures expressing αORF and βkinase were grown to a turbidity of 0.5 or 1.0, respectively, for infiltration.

As shown in Figure 4A, ion leakage levels induced by transient expression of the NbMAPKKKβ kinase domain or full-length NbMAPKKKγ were lower in *NbMAPKKKα*-silenced plants than in VIGS vector-only control plants. Cell death induced by both NbMAPKKKβ and NbMAPKKKγ was consistently significantly suppressed in *NbMAPKKKα*-silenced plants compared to control plants (data not shown). This result suggests that NbMAPKKKα either acts downstream of both NbMAPKKKβ and NbMAPKKKγ in cell death induction or plays an essential role in codependent activation of both NbMAPKKKβ and NbMAPKKKγ. In contrast, the ion leakage levels induced by transient expression of full-length NbMAPKKKα or NbMAPKKKγ were similar in *NbMAPKKKβ*-silenced plants and control plants (Figure 4B), suggesting that NbMAPKKKα and NbMAPKKKβ are not codependent in their activation; rather, NbMAPKKKβ functions upstream of NbMAPKKKα in the cell death signaling pathway. This result led us to postulate that NbMAPKKKβ functions upstream of NbMAPKKKγ. As expected, in *NbMAPKKKγ*-silenced plants, cell death and the ion leakage level induced by the transient overexpression of the NbMAPKKKβ kinase domain were significantly compromised compared to control plants (data not shown and Figure 4C, respectively). Cell death and the ion leakage induced by transient overexpression of NbMAPKKKα, however, were similar in *NbMAPKKKγ*-silenced and control plants (data not shown and Figure 4C, respectively), confirming that NbMAPKKKβ functions upstream of NbMAPKKKγ and that NbMAPKKKγ functions upstream of NbMAPKKKα. In this epistasis analysis, ion leakage levels were closely associated with the intensity of cell death at all combinations of these genes. Thus, these three MAPKKK genes form a linear signaling pathway leading to PCD in which NbMAPKKKβ and NbMAPKKKα function as the furthest upstream and downstream components, respectively, of the three MAPKKK components.

## Discussion

In our previous study [19], we showed that a MAPK cascade including NbMAPKKKα and NbMEK2 is required for both the PCD-associated systemic necrosis induced by PlAMV-Li1 and the *Rx*-mediated HR against potato virus X. Several studies have demonstrated that at least two MAPK cascades are involved in plant responses to various environmental stimuli [8,12]. The great diversity of plant MAPKKK genes is assumed to underlie the ability of plants to mount specific signaling responses to various environmental stimuli [15,16]. Therefore, to better understand the functions of MAPKKKs in the induction of PCD, we performed a comparative functional analysis of *N. benthamiana* MAPKKK genes. In the previous and present study, we demonstrated that three of these genes, *NbMAPKKKα*[19], *NbMAPKKKβ*, and *NbMAPKKKγ* (and/or its homologs), function as positive regulators of PlAMV-Li1-induced PCD and are not functionally redundant in the cell death signaling pathway. In the VIGS experiments, not only *NbMAPKKKγ* but also its homologous gene(s) were presumably knocked down in TRV-*NbMAPKKKγ*-infected plants. However, only one gene in the A2 subgroup was obtained from the cloning experiments, suggesting that *NbMAPKKKγ* homologous gene(s), which carries almost the same sequence as *NbMAPKKKγ* itself, must be effectively knocked down in TRV-*NbMAPKKKγ*-infected plants. In addition, overexpression of the *NbMAPKKKγ* full-length ORF and kinase domain could induce rapid cell death. Therefore, NbMAPKKKγ itself could play an important role in the cell death signaling pathway.

We also explored the functional relationship among these three MAPKKK genes by performing an epistasis experiment based on the assumption that if signaling protein A functions upstream of signaling protein B, signaling by activated A will be suppressed by silencing of B expression, whereas signaling by activated B will not be suppressed by silencing of A expression. Our results (Figure 4) demonstrate that NbMAPKKKα, NbMAPKKKβ, and NbMAPKKKγ, which represents NbMAPKKKγ and/or its homologous gene(s), form a linear signaling pathway leading to cell death induction in which NbMAPKKKβ acts upstream of NbMAPKKKγ and NbMAPKKKγ acts upstream of NbMAPKKKα. Results of a similar epistasis analysis have suggested that an NPK1/MEK1/NTF6 MAPK cascade functions downstream of a MAPKKKα/MEK2/SIPK MAPK cascade in the induction of PCD in plants [8]. These results support the possibility that NbMAPKKKα, NbMAPKKKβ, NbMAPKKKγ, and NPK1 initiate four distinct MAPK cascades that are coordinately involved in plant cell death. Alternatively, different sets of MAPK cascades might be involved in cell death signaling pathways induced by different plant–microbe interactions. To investigate this issue, further research is needed to determine whether NbMAPKKKβ and NbMAPKKKγ participate in the *N* gene-mediated HR against TMV or in the *Pto*-mediated HR against Pst.

In our experiments, the transient overexpression of the NbMAPKKKε2 kinase domain by agroinfiltration did not induce cell death (Figure 2A). However, Melech-Bonfil and associates have shown that the tomato homolog of NbMAPKKKε2, SlMAPKKKε, is required for PCD induction in the HR against Gram-negative bacterial pathogens [9]. Furthermore, they showed that the transient overexpression of the tomato SlMAPKKKε kinase domain in *N. benthamiana* induces pathogen-independent cell death. A comparison between the amino acid sequences of the kinase domains of NbMAPKKKε2 and SlMAPKKKε indicated only three amino acid differences. These three residues are not highly conserved and are not predicted to be essential for plant kinase catalytic activity, so explaining this functional difference solely by the amino acid sequence level is difficult. Although at first glance, our results seem to contradict theirs, the results cannot be directly compared because three factors in our transient overexpression experiments differed from theirs: promoters, *Agrobacterium* strains, and species origin of the homologous genes. These three factors, which could influence the expression levels of overexpressed proteins, are critical for the execution of cell death elicited by proteins possessing cell death-inducing activity [27,28]. Moreover, the amount of overexpressed NbMAPKKKε2 kinase domain in our experiment was much more than the amounts of NbMAPKKKβ kinase domain and its K → M mutant or NbMAPKKKγ kinase domain that can induce cell death and was comparable to the amount of the NbMAPKKKγ kinase domain K → M mutant. Therefore, NbMAPKKKε2 might be a less potent inducer of cell death than other MAPKKKs.

Our suggestion that several MAPK cascades function in a linear signaling pathway in the induction of cell death raises a concern about their roles in plant defense responses. To prevent pathogen invasion, plants commonly employ two types of receptors [NBS-LRR (nucleotide-binding site and leucine-rich repeat) receptors and RLK (receptor-like kinase)-type receptors] [1] to sense multiple PAMPs or effectors derived from biotrophic and necrotrophic pathogens. When plants perceive pathogens, the transcription of a common set of genes that act against various types of pathogens is activated [29,30], subsequently producing many defense responses, including cell death and the production of low-molecular-weight signaling compounds such as ethylene and reactive oxygen species (ROS). Activation of the MAPKKKα/MEK2/SIPK cascade can induce production of ethylene and ROS [31,32]. NPK1, which initiates another MAPK cascade, is activated by the ROS produced by the MAPK cascade initiated by MAPKKKα [26]. Similarly, the alfalfa MAPKKK MsOMTK1 is activated by hydrogen peroxide [6]. These results suggest that compounds induced by an upstream-acting MAPK cascade function as signaling molecules that activate a downstream-acting MAPK cascade. In addition, they suggest that the involvement of several MAPK cascades in defense responses enables plants to activate various responses simultaneously or coordinately to combat various types of pathogens.

## Conclusions

In this study, we demonstrated that three MAPKKKs in *N. benthamiana* form a linear signaling pathway leading to PCD, implying that the involvement of multiple MAPK cascades in plant defense responses enables plants to exhibit various reactions simultaneously. Further analysis to identify downstream MAPKK and MAPK genes directly phosphorylated by NbMAPKKKβ and NbMAPKKKγ should help clarify the regulatory mechanisms of cell death involving these MAPKKK genes. Furthermore, to elucidate the mechanisms underlying the sequential activation of each MAPK cascade during plant defense responses, the upstream components or chemical compounds that directly activate these MAPKKKs must be identified.

## Methods

### Plant materials and virus isolate

*N. benthamiana* plants were grown in a growth chamber at 25 °C. To virally induce PCD, plants were inoculated with the binary vector pLi1, which contains the full-length cDNA of the Li1 isolate of plantago asiatica mosaic virus (PlAMV) downstream of the cauliflower mosaic virus 35 S promoter [33].

### Cloning of MAPKKK genes from *N. benthamiana*

To retrieve partial MAPKKK cDNA sequences, a BLAST search was performed using the amino acid sequences of the kinase domains of the *Arabidopsis* MAPKKK genes *AtMEKK1*, *AtMAPKKKγ*, and *AtMAPKKKε1* as queries against the *N. benthamiana*, *N. tabacum*, and *S. lycopersicum* databases of the Gene Index Project at the Computational Biology and Functional Genomics Laboratory Web site (http://compbio.dfci.harvard.edu/tgi/cgi-bin/tgi/Blast/index.cgi) and the tobacco BY-2 EST clone database of the RIKEN BioResource Center (http://www.brc.riken.go.jp/lab/epd/blast/index.shtml). The consensus nucleotide sequences for the retrieved partial cDNA sequences and query sequences were used to design a pair of specific primers for each of the three MAPKKK genes. Each pair of specific primers was used for RT-PCR amplification of a cDNA fragment from total RNA extracted from *N. benthamiana* leaves. The RT-PCR products were gel-purified and cloned into the pGEM-T easy vector (Promega, Madison, WI, USA) for sequencing. New primers were then designed based on the regions of the obtained cDNA sequences that were nonhomologous to the query sequences (to prevent nonspecific amplification) and used for specific 5′- and 3′-rapid amplification of cDNA ends (RACE) using a GeneRacer Kit (Invitrogen, Carlsbad, CA, USA) according to the standard protocol provided by the manufacturer. The sequences of primers used in this study are listed in Table 1. cDNA sequences of these MAPKKK genes were determined from at least three independent clones.

**Table 1:** Primers used in this study

**Name**	**Sequence (5’-3’)**	**Comments**
NbTC9992-1F	GCTGTCAAAGAAGTGTCATTA	Specific primer for TC9992
NbTC9992-1280R	ACCGTTTATTAATCACTATATTGC	Specific primer for TC9992
NtBP1333-1 F	CTTAATGGGCAAGCAGCTAATC	Specific primer for BP133312
NtBP1333-447R	TCAAGATTGTATGTTGTCTGCTC	Specific primer for BP133312
LeBI9315-123F	GTTGCAATTAAACAAGTTTCTCTGGA	Specific primer for BI931567
LeBI9315-658R	GGCTGAAGATCATAGTACGG	Specific primer for BI931567
NbTC9992-458R-5RACE	GCTTGTCCATAGCCTTGGTTCTTCCT	5’-RACE for TC9992
NbTC9992-49R-5RACE	TTTGCCTTCCCCCATCGCCTTGAT	5’-RACE for TC9992
NbTC9992-F1	GGATTGGAAAGGGGGAACCT	Sequencing
NtMAP3Kb-566F	TCCGCCGGTCATGTCACT	Sequencing
NtBP1333-199R-5RACE	ACATGGCTGCAGCTGCTTCATATTC	5’-RACE for BP133312
NtBP1333-91R-nested	TACTATCCTTCTGCATAACTGACTGCAA	5’-RACE for BP133312
NtBP1333-333F-5RACE	GAGAATCTCTCATCAGCCAGATGTTC	3’-RACE for BP133312
NtBP1333-375F-nested	CAAACCTGTTGGTGGGGTACGAAT	3’-RACE for BP133312
NtBP1333-F1	AGACGCGCATAATTCGCATC	Sequencing
NtBP1333-R1	TGAGCTCTCGTTTGGTAATAAG	Sequencing
LeBI9315-507R-5RACE	CATCTGCCTCTGTCAACTTTGTTGCA	5’-RACE for LeBI931567
LeBI9315-165R-nested	CCTCCTGAGCAATATTCTCCAGAGA	5’-RACE for LeBI931567
LeBI9315-482F-3RACE	TGCAACAAAGTTGACAGAGGCAGATG	3’-RACE for LeBI931567
LeBI9315-564F-nested	ATGTCGGGAGTATGTGCTGCATCTG	3’-RACE for LeBI931567
LeBI9315-F1	CTGAGAAAGTTCTTGCAAACG	Sequencing
LeBI9315-F2	GAACAGATGAATCAGAAGATG	Sequencing
LeBI9315-F3	AAAGCACTCCATATAAACACAG	Sequencing
LeBI9315-R1	GGTATACATCAAGTCCACCAT	Sequencing
LeBI9315-R2	GAGGGAGTATGCTCTCATG	Sequencing
Kp-NbMAPKKKa-1F	GGGGTACCGAATGCCTGCTTGGTGGGGAA	Full-length ORF of NbMAPKKKα
Xh-NbMAPKKKa-1836R	GGCTCGAGTGCTAAAGAATTGGTCTTAGTTTTG	Full-length ORF of NbMAPKKKα
Kp-NbMAPKKKb-1F	CCGGTACCGAATGCATCGATTGCCAGGAATTTTTGC	Full-length ORF of NbMAPKKKβ
EcV-NbMAPKKKb-1695R	GGGATATCTTTAAAGCCTCTTGCCCAGATTTTG	Full-length ORF of NbMAPKKKβ
Kp-NbMAPKKKg-1F	GGGGTACCGAATGCGTTGGTGGCAGAACG	Full-length ORF of NbMAPKKKγ
Xh-NbMAPKKKg-1956R	GGCTCGAGTGCTACCTCTCTAGAGATAAACG	Full-length ORF of NbMAPKKKγ
Kp-NbMAPKKKbkinase-F	CCGGTACCGAATGTACTGGGACAAAGGTGATCT	Kinase domain of NbMAPKKKβ
EcV-NbMAPKKKbkinase-R	GGGATATCTTTACACAAAAGGATGCTCCAAGA	Kinase domain of NbMAPKKKβ
Kp-NbMAPKKKgkinase-F	GGGGTACCGAATGTGGCAAAAAGGGAAGCTTATTG	Kinase domain of NbMAPKKKγ
Xh-NbMAPKKKgkinase-R	GGCTCGAGTGTTACATAAATCGATGTTCCAATAAC	Kinase domain of NbMAPKKKγ
Kp-NbMAPKKKekinase-F	GGGGTACCGAATGAAATATATGCTCGGAGATGAG	Kinase domain of NbMAPKKKε2
Xh-NbMAPKKKekinase-R	GGCTCGAGTGTTATATCCATGGATGTGAAAGTAG	Kinase domain of NbMAPKKKε2
NbMAPKKKb-K381M-F	TTTTGCTGTCATGGAAGTGTCATTACTTGATCA	K → M mutant of NbMAPKKKβ
NbMAPKKKb-K381M-R	ATGACACTTCCATGACAGCAAAAAAGAAACCG	K → M mutant of NbMAPKKKβ
NbMAPKKKg-K374M-F	CTGGAGCTTTATGTGCGATGATGGAAGTTGAATT ATTACCGGA	K → M mutant of NbMAPKKKγ
NbMAPKKKg-K374M-R	TCCGGTAATAATTCAACTTCCATCATCGCACATAA AGCTCCAG	K → M mutant of NbMAPKKKγ
NbMAPKKKa-255F	GGTTGTTTTGGGATGTGGGGTCAG	Real-time RT-PCR for NbMAPKKKα
NbMAPKKKa-393R	CAGTGGGCTCAACCTATTATCGCC	Real-time RT-PCR for NbMAPKKKα
NbMAPKKKb-1179F	CACAAGGCAGATTTTACATGGTTTG	Real-time RT-PCR for NbMAPKKKβ
NbMAPKKKb-1286R	AGCTTGACCGATCCGTTAGCA	Real-time RT-PCR for NbMAPKKKβ
NbMAPKKKg-903F	CCGTGAGTGTAGTGCTCAGGGTAA	Real-time RT-PCR for NbMAPKKKγ
NbMAPKKKg-984R	TGCCGTAGGCTGCTGTGATG	Real-time RT-PCR for NbMAPKKKγ
Nb18S-193F	ATACGTGCAACAAACCCCGAC	Real-time RT-PCR for Nb18S rRNA
Nb18S-280R	TGAATCATCGCAGCAACGG	Real-time RT-PCR for Nb18S rRNA

ORF and motif analyses were performed using the ORF Finder (http://www.ncbi.nlm.nih.gov/gorf/gorf.html) and CD-Search (http://www.ncbi.nlm.nih.gov/Structure/cdd/wrpsb.cgi) programs, respectively. Phylogenetic analysis was performed using MEGA3.1 based on a multiple alignment created using ClustalW. The MAPKKK gene sequences used in phylogenetic analysis are listed in the GenBank database under the following accession numbers: *AtMEKK1* (NM_116919), *AtMEKK2* (NM_116917), *AtMEKK3* (NM_116916), *AtMEKK4* (NM_117272), *BnMAPKKKβ1* (AJ010093), *AtANP1* (NM_100771), *AtANP2* (NM_104370), *AtANP3* (NM_111477), *NtNPK1* (D26601), *AtMAPKKKγ* (NM_126084), *AtYODA* (AY357949), *NbMAPKKKα* (AY500155), *AtMAPKKKα* (NM_179472), *AtMAPKKKε1* (NM_112199), *AtMAPKKKε2* (NM_111677), *MsOMTK1* (AJ575100), *BnMAPKKKα1* (AJ010091), *BnMAPKKKε1* (AJ238845), *SlMAPKKKε* (GU192457), and *NbMAPKKKε* (GU205153). *Homo sapiens HsMEKK1* (AF042838) was used as an outgroup.

### Construction of plasmids for transient expression of wild-type and mutant MAPKKKs and MAPKKK kinase domains and agroinfiltration

The coding regions of *NbMAPKKKα*, *NbMAPKKKβ*, and *NbMAPKKKγ* were amplified by RT-PCR using primer pairs Kp-NbMAPKKKa-1F/Xh-NbMAPKKKa-1836R, Kp-NbMAPKKKb-1F/EcV-NbMAPKKKb-1695R, and Kp-NbMAPKKKg-1F/Xh-NbMAPKKKg-1956R, respectively. The kinase domains of *NbMAPKKKβ*, *NbMAPKKKγ*, and NbMAPKKK*ε2* were amplified using primer pairs Kp-NbMAPKKKbkinase-F/EcV-NbMAPKKKbkinase-R, Kp-NbMAPKKKgkinase-F/Xh-NbMAPKKKgkinase-R, and Kp-NbMAPKKKekinase-F/Xh-NbMAPKKKekinase-R, respectively.

Loss-of-function mutants of NbMAPKKKβ and NbMAPKKKγ were obtained by substituting methionine for the essential lysine in the ATP-binding site in the kinase domain. These mutants were obtained by site-directed mutagenesis using primers NbMAPKKKb-K381M-F and NbMAPKKKb-K381M-R for NbMAPKKKβ and NbMAPKKKg-K374M-F and NbMAPKKKg-K374M-R for NbMAPKKKγ. Each PCR-amplified MAPKKK gene fragment was subcloned into the pEarleyGate 203 vector under 35 S promoter [34] via the pENTA entry vector [35] with LR Clonase II Enzyme Mix (Invitrogen). GUS gene was also subcloned into the pEarleyGate 203 vector under the 35 S promoter. These plasmid vectors were transformed into *Agrobacterium tumefaciens* strain EHA105. Agroinfiltration was performed as previously described [27].

### Construction of VIGS vectors and VIGS

VIGS was performed as described previously using pTV:00 derivatives and pBintra6 [17]. NbMAPKKKα silencing was induced using pTV:*NbMAPKKKα*[19]. For NbMAPKKKβ and NbMAPKKKγ silencing, the 390-bp KpnI–PvuII fragment of the 5′-terminal region of the PCR-amplified full-length NbMAPKKKβ ORF and the 576-bp KpnI–EcoRV fragment of the PCR-amplified full-length NbMAPKKKγ ORF were introduced into pTV:00 in the antisense direction to generate pTV:*NbMAPKKKβ* and pTV:*NbMAPKKKγ*, respectively. The NbMAPKKKβ ORF was amplified using primers Kp-NbMAPKKKb-1 F and EcV-NbMAPKKKb-1695R, and the NbMAPKKKγ ORF was amplified using primers Kp-NbMAPKKKg-1 F and Xh-NbMAPKKKg-1956R.

### Real-time RT-PCR

The methods used for total RNA isolation, cDNA synthesis, and quantitative analysis of gene expression were the same as those previously described [18]. Expression of 18 S rRNA was used as the normalizer. The primers used for real-time RT-PCR were NbMAPKKKa-255F and NbMAPKKKa-393R for NbMAPKKKα, NbMAPKKKb-1179F and NbMAPKKKb-1286R for NbMAPKKKβ, NbMAPKKKg-903F and NbMAPKKKg-984R for NbMAPKKKγ, and Nb18S-193F and Nb280R for Nb18S rRNA.

## Abbreviations

HR: Hypersensitive response; MAPK: Mitogen-activated protein kinase; MAPKK: MAPK kinase; MAPKKK: MAPKK kinase; PCD: Programmed cell death; PlAMV: plantago asiatica mosaic virus; Pst: *Pseudomonas syringae* pv. *tomato*; TMV: tobacco mosaic virus.

## Authors’ contributions

MH, KK, YY and SN designed the experiments. MH and KK performed the experiments and analyzed the data together with KM, YO, YY and SN. TS, KI and YT contributed new reagents and analytic tools. MH, KK and SN wrote the paper. All authors discussed the results and approved the final manuscript.

## Supplementary Material

Additional file 1 **Figure S1.** Southern blot analysis of **A**) *NbMAPKKKβ* and **B**) *NbMAPKKKγ* using kinase domain-specific DNA probes. DNA probes were generated by using the PCR DIG Probe Synthesis Kit (Roche, Basel, Switzerland) according to the manufacturer’s instructions. Each lane was loaded with 5 μg of total genomic DNA digested with each restriction enzyme. Click here for file

Additional file 2 **Figure S2.** Ion leakage of GUS-infiltrated areas. An *Agrobacterium* strain expressing the GUS gene using the 35 S promoter was infiltrated at the following turbidities: 0.05, 0.5, and 1.0. Click here for file
