# Supplementary figures and images for: Identification of three MAPKKKs forming a linear signaling pathway leading to programmed cell death in Nicotiana benthamiana
Source: BMC Plant Biol. 2012 Jul 8;12:103. doi: 10.1186/1471-2229-12-103 (PMC3507812; doi:10.1186/1471-2229-12-103)

A

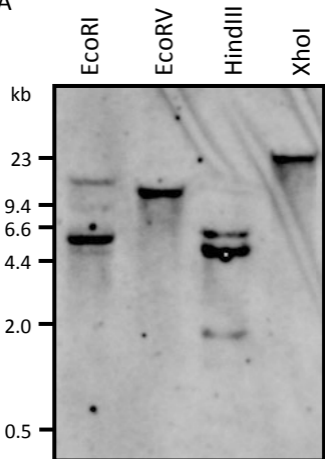

B

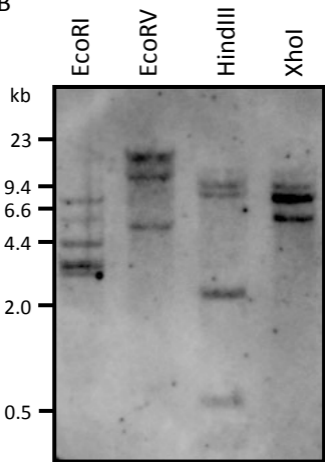

Supplement: Additional file 1 — Figure S1. Southern blot analysis of A) NbMAPKKKβ and B) NbMAPKKKγ using kinase domain-specific DNA probes. DNA probes were generated by using the PCR DIG Probe Synthesis Kit (Roche, Basel, Switzerland) according to the manufacturer’s instructions. Each lane was loaded with 5 μg of total genomic DNA digested with each restriction enzyme. [file 1471-2229-12-103-S1.pdf]

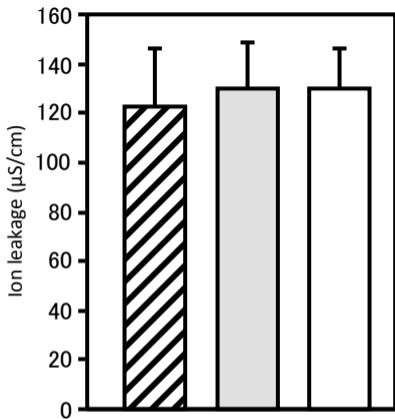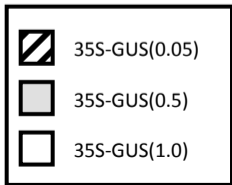

Supplement: Additional file 2 — Figure S2. Ion leakage of GUS-infiltrated areas. An Agrobacterium strain expressing the GUS gene using the 35 S promoter was infiltrated at the following turbidities: 0.05, 0.5, and 1.0. [file 1471-2229-12-103-S2.pdf]
